# Microfluidic System-Based Quantitative Analysis of Platelet Function through Speckle Size Measurement

**DOI:** 10.3390/biom14060612

**Published:** 2024-05-23

**Authors:** Jong Hyeok Han, Inkwon Yoon, Hee-Jae Jeon

**Affiliations:** 1 Department of Mechanical and Biomedical Engineering, Kangwon National University, Chuncheon 24341, Republic of Korea; 2 Department of Smart Health Science and Technology, Kangwon National University, Chuncheon 24341, Republic of Korea

**Keywords:** platelet aggregation, laser speckle, speckle size, platelet activator

## Abstract

Platelets play essential roles in the formation of blood clots by clumping with coagulation factors at the site of vascular injury to stop bleeding; therefore, a reduction in the platelet number or disorder in their function causes bleeding risk. In our research, we developed a method to assess platelet aggregation using an optical approach within a microfluidic chip’s channel by evaluating the size of laser speckles. These speckles, associated with slowed blood flow in the microfluidic channel, had a baseline size of 28.54 ± 0.72 µm in whole blood. Removing platelets from the sample led to a notable decrease in speckle size to 27.04 ± 1.23 µm. Moreover, the addition of an ADP-containing agonist, which activates platelets, resulted in an increased speckle size of 32.89 ± 1.69 µm. This finding may provide a simple optical method via microfluidics that could be utilized to assess platelet functionality in diagnosing bleeding disorders and potentially in monitoring therapies that target platelets.

## 1. Introduction

Platelets are important components of pathological thrombosis, and normal hemostasis and abnormalities in platelet count are considered a type of bleeding disorder. Both thrombocytopenia (a low platelet count) and thrombocytosis (a high platelet count) can lead to issues such as bleeding or clotting problems [1,2,3]. Thus, bleeding disorders related to platelet number are key issues before monitoring and surgery of the patient’s condition [4,5,6,7,8]. Platelet aggregation leads to the formation of blood clots and can cause blockage of small capillary vessels, acute trauma, and surgery (blocking the small capillary vessel in acute trauma, surgery procedures, and many illnesses), which can cause life-threatening bleeding [9,10]. Thus, monitoring platelet function is crucial for preventing severe bleeding and for detection and treatment in the case of thrombotic conditions [8,11,12].

An indicator of platelet aggregation status can be obtained by using a conventional coagulation test and light transmission aggregometry (LTA), which are closely associated with the measurement of stopped bleeding time and the light transmission intensity variation [13,14]. Unfortunately, owing to the long reporting time and difficulty of standardization, conventional coagulation tests have limited clinical utility [15,16]. Thus, several methods and devices are introduced for measuring platelet function, such as impedance aggregometry, a platelet function analyzer (PFA-100), and flow cytometry [17,18]. However, the measurement performance of these systems requires a relatively large blood volume of 1 mL and a measurement time of 15 min [12].

More recently, several microfluidic systems using smaller sample volumes, particularly those qualifying platelet adhesion using shear rate distribution and specifically designed microfluidic devices, have been proposed to measure the function of platelets in whole blood [12,19,20,21]. However, the proposed system requires a complicated fabrication process, a shear-inducing system, and a high-speed sCMOS camera.

While some previous studies have explored the relationship between speckle size and platelet function under static conditions [22,23], our study introduces a significant advancement by incorporating a microfluidic system to measure speckle size dynamics, thereby simulating more accurate physiological blood flow conditions. This innovative approach not only mitigates potential biases introduced by static environments but also enables precise control over experimental variables, enhancing the reliability and applicability of our findings. In this study, we prepared two distinct sample types to assess platelet number and developed a microfluidic system equipped with laser speckle imaging, enabling a rapid and straightforward assay for simultaneous measurement of speckle size and contrast. Using our system, we analyzed variations in speckle size and contrast that were directly correlated with platelet function, influenced by ADP agonists, across both whole blood and platelet-poor samples, offering insights into platelet activation mechanisms.

## 2. Materials and Methods

### 2.1. Measurement of Speckle Size and Contrast

When the laser light illuminates the rough surface, a speckle pattern is generated owing to random interference. The time series of the 2D speckle pattern images was captured using an sCMOS camera to calculate the speckle size and contrast. The average speckle size was calculated by normalizing the autocovariance function of the speckle intensity [22,24,25]. The average speckle size was calculated using Equation (1).

(1) $$s=\frac{R_{i}\left(x,y\right)-{\langle I(x,y)\rangle }^{2}}{\langle {I(x,y)}^{2}\rangle -{\langle I(x,y)\rangle }^{2}}\;where\;R_{i}\left(x,y\right)=\langle I(x_{i}y_{i})\cdot I(0,0)\rangle ,$$

where *s* is the speckle size, $R_{i}(x,y)$ is the autocorrelation function, and $I\left(x,y\right)$ is the intensity recorded at a pixel of the sCMOS. The level of blurring was quantified by the speckle contrast, defined as the ratio of the standard deviation to the intensity [26].

(2) $$C=\frac{\sigma }{\left[I\right]},$$

where [*I*] and *σ* represent the mean value of light intensity and standard deviation, respectively. If the scattering particles move over the surface of the microfluidic device, the speckle pattern fluctuates and becomes blurred, resulting in a decrease in the speckle contrast of the grid. Thus, we measured the speckle size and contrast between whole and platelet-poor blood using a square-shaped microfluidic chip.

### 2.2. Speckle Size Measurement System

**Figure 1:**
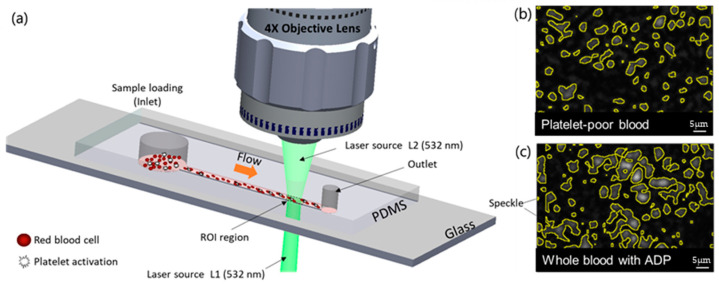
Schematic of the setup used to measure the speckle size of platelet-poor and whole blood. (**a**) The green (*λ* = 532 nm) laser light passes through a microchannel, and the ROI is captured by the objective lens of the microscope. (**b**,**c**) show the laser speckle images for analyzing speckle size between platelet-poor and whole blood with ADP (ROI: region of interest; PDMS: polydimethylsiloxane).

Figure 1 shows the speckle size measurement system. A green laser (*λ* = 532 nm) with an output power of 50 mW (PSU-III-LCD, Changchun New Industries Optoelectronics Technology Co., Ltd., Jilin, China) passes through the microfluidics chip. A 4X objective lens (Plan N 4C, NA 0.1, Tokyo, Japan) is focused on the surface of blood flow in the microchannel and captures speckle images from the CMOS camera (Neo 5.5 sCMOS, Andor Technology Ltd., Belfast, UK) with an exposure time of 0.8 ms and a frame rate of 1250 fps. A linear polarizer with a 90° angle, an aperture with a diameter of 5 mm, and a tube lens (focal length of 180 mm) were placed in front of the sCMOS camera to improve contrast.

### 2.3. Microparticle Preparation and Blood Sample Preparation

We obtained 1 mL of blood from a rat tail by intravenous injection using a 23 G needle under isoflurane anesthesia and used over 11 male Sprague–Dawley rats (12–13 weeks old with body weights between 250 and 280 g) at an interval of at least two weeks. All animal handling followed the guidelines of the Institutional Animal Care and Use Committee (IACUC) of the Gwangju Institute of Science and Technology, Korea (# GIST-2019-015). Blood samples were collected in citrate tubes (#363083, 9NC 0.109 M Buffered Trisodium Citrate, BD Vacutainer, Franklin Lakes, NJ, USA). Whole blood was centrifuged (Velocity 18R, Dynamica Scientific Ltd., Livingston, UK) at 3000 rpm for 5 min to prepare platelet-poor blood and remove platelets [27,28]. The buffy coat of the whole blood sample was carefully removed using a micropipette. For all of our experiments, the hematocrit was maintained at 40–45%, and all experiments were conducted within 1 h of preparing the whole blood sample. Additionally, we used 1 μm microparticles (#08168, Sigma Aldrich, St. Louis, MO, USA) to measure the speckle size variation depending on particle concentration. Each particle concentration was prepared using diluted deionized (DI) water: 1:10 (0.13% or 9.1 × 10^9^ particles/mL), 3:10 (0.42% or 2.73 × 10^10^ particles/mL), or 5:10 (0.63% or 4.55 × 10^10^ particles/mL).

### 2.4. Fabrication of Microfluidic Device and System Operation

**Figure 2:**
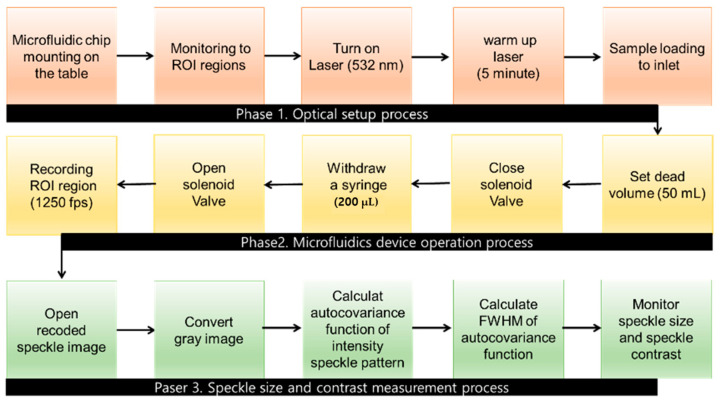
Schematic and detailed flow chart of the procedure used to measure the speckle size. The three steps include optical setup, microfluidics device operation, and speckle size measurement.

We designed a channel (45 mm in length, 45 μm in height, and 1 mm in width) to fabricate a master mold and employed standard soft photolithographic techniques, allowing us to capture images near the outlet where flow rate variations are most significant [29]. We prepared the PDMS slab using a fabrication process that included mixing, air removal, and curing. The prepared PDMS (Sylgard 184 A/B, DowWhitech, Goyang, Republic of Korea) slab was bonded to a cover glass after oxygen plasma treatment. The overall process of microfluidic operation is shown in Figure 2. We used a large dead volume to relieve pressure fluctuations (50 mL) to relieve pressure fluctuations and prevent rapid displacement caused by internal pressure thus maintaining a controlled environment within the channel. A pulling volume of 200 µL was set to ensure minimum pressure for movement towards the outlet, using the withdrawal mode of the syringe pump during the closing of the solenoid valve. We chose a location near the outlet reservoir with a 1 mm diameter at the end of the channel for the sCMOS camera and waited for the laser to warm up (5 min) to monitor the region of interest (ROI; 0.8 × 3.3 mm). The solenoid valve was opened after the blood sample was introduced into the inlet. When the blood sample passed through the microfluidic channel and arrived at the initial ROI, the sCMOS camera began capturing it.

### 2.5. Flow Cytometry Measurement for Counting of Platelet Number

To evaluate the sample preparation accuracy, we measured the platelet number using FACSCalibur (BD FACSalibur, Becton Dickinson Immunocytometry Systems, San Jose, CA, USA) with CELLQuest. Whole blood (500 µL) was pipetted into a TruCOUNT tube for flow cytometry platelet analysis. The amount of antibody (anti-CD61 and anti-CD41) added for sample preparation was according to the manufacturer’s recommendations [30,31]. The sample mixture was gently mixed and incubated for 15 min at room temperature (18–22 °C) in the dark. For data acquisition and analysis, a sample containing the stained platelets was added to the flow cytometer. The sample was focused on the flow of one cell in the channel. The laser beam passed through the channel, and the scattered light was detected.

### 2.6. Confirmation of Platelet Aggregation by DIOC6 Staining

To confirm the platelet aggregate size, the DIOC6 staining method was applied to a microfluidic chip. We used a solution of 3′-dyhexyloxacarbocyanine iodide (DiOC6) (#53213-82-4, Sigma Aldrich) and prepared a 1 mM solution distilled in DI water. Whole blood was collected in a separate citrated tube; one citrate tube from the buffy coat region was removed by centrifugation (3000× *g* for 10 min), and the remaining tube remained as it was. A 2 µL DiOC6 solution (1 mM) was added to each citrated tube. The samples were incubated for 10 min at 37 °C after gentle mixing [32]. After filling the samples, the microfluidic channel was illuminated with green LED light (#TB-X6-RCP, Live Cell Instrument, Namyangju, Republic of Korea), and fluorescent images were captured using a fluorescence microscope with a 20× objective lens.

## 3. Results

**Figure 3:**
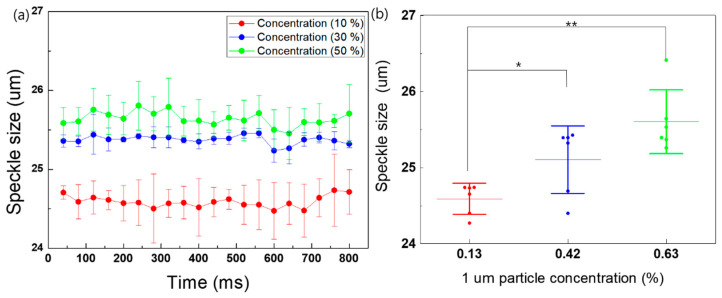
Speckle size comparison at varying particle concentrations of 10%, 30%, and 50% (*n* = 5). (**a**) Speckle size calculated during the aggregation process over time and (**b**) averaged speckle size at a 1 um particle concentration with experimental measurements for the speckle pattern. Each particle concentration was prepared using 1 um diameter with a 1.25 particle concentration and diluted with 1, 3, and 10 mL of DI water. * indicates *p* < 0.05, and ** indicates *p* < 0.01.

We compared the speckle size with respect to the difference in microparticle concentration (Figure 3). The polystyrene microsphere had a diameter of 1 μm. The final solution concentration was determined by diluting it with DI water (10%, 30%, and 50%). At the highest particle concentration (50%), the speckle size increased significantly. The speckle size at each concentration measured approximately 24.57 ± 0.2, 25.09 ± 0.39, and 25.61 ± 0.39 um, respectively, with linear regression (y = 0.57x + 23.92). Platelet counts were measured to confirm the number of platelets in the whole and platelet-poor blood samples (n = 10). The whole blood platelet count was 1244 ± 0.06 × 10^3^/µL, and that of the platelet-poor blood counted by removing the platelets using centrifugation methods was 13.06 ± 0.85 × 10^3^/µL.

**Figure 4:**
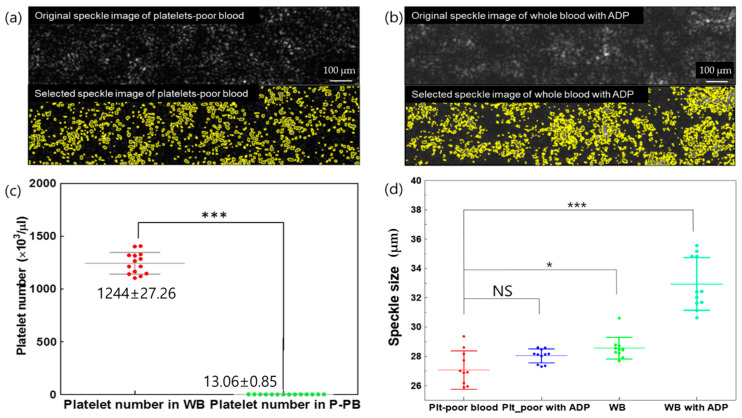
Speckle size difference based on the addition of ADP to whole and platelet-poor blood. (**a**) Original speckle image and processed speckle image between platelet-poor and (**b**) whole blood depending on the addition of ADP obtained using the ImageJ selected function. (**c**) Difference in platelet numbers between platelet-poor and whole blood via flow cytometry. The speckle size variation during the aggregation process was due to the different conditions applied to the whole and platelet-poor blood in the figure (**c**,**d**). NS indicates no significant difference; * indicates *p* < 0.05; and *** indicates *p* < 0.001.

We measured the speckle size by monitoring the time series of speckle patterns between platelet-poor and whole blood. Figure 4a,b shows the speckle pattern of the original image (top) and the selected image of the speckle pattern (bottom). The addition of ADP significantly increases the speckle size. We calculated the speckle size using the autocovariance function; the speckle size increased depending on the platelet number and ADP agonist (Figure 4c,d). The measured speckle sizes were 27.04 ± 1.24, 28.02 ± 0.46, 28.54 ± 0.72, and 32.89 ± 1.69 um.

**Figure 5:**
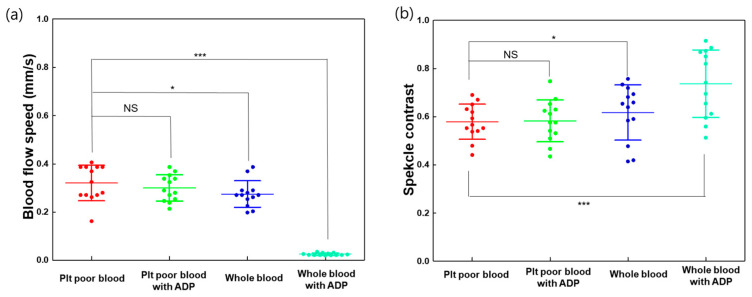
Blood flow speed and speckle contrast difference in response to ADP agonist between whole and platelet-poor blood. (**a**) Blood sample flow speed with platelet activator (ADP) and (**b**) effects of ADP on speckle contrast. A speckle contrast value close to 1 indicates a static condition, and a value close to 0 indicates the fast motion of scatters, such as a moving blood sample. NS indicates no significant difference; * indicates *p* < 0.05, and *** indicates *p* < 0.001. Plt: platelet.

The blood flow speed was recorded within the ROI as the touch-down time of the blood from the start to the end of the ROI, with a length of 0.8 mm. The migration speed of the blood sample decreased as platelet reactivity increased with the addition of ADP. Figure 5a shows the blood flow speed difference between whole and platelet-poor blood in response to the ADP agonist. The blood flow speeds were 0.32 ± 0.07, 0.3 ± 0.05, 0.27 ± 0.05, and 0.025 ± 0.005 mm/s for the corresponding platelet-poor blood, platelet-poor blood with ADP, whole blood, and whole blood with ADP, respectively. The speckle contrast was also measured using Equation (2). The speckle contrast increased in response to the platelet activity with the addition of ADP. Platelet-poor and whole blood without ADP exhibited speckle contrasts of 0.58 ± 0.070 and 0.62 ± 0.08, respectively, while platelet-poor and whole blood with ADP exhibited 0.58 ± 0.08 and 0.73 ± 0.13, respectively.

**Figure 6:**
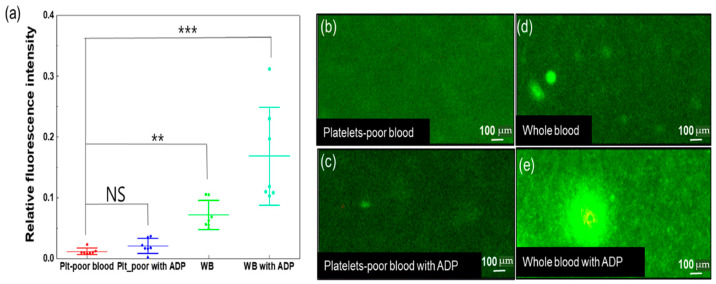
Platelet aggregation based on the addition of ADP between platelet-poor and whole blood. (**a**) Measurement of the relative fluorescence intensity (**b**–**e**) captured from sCMOS camera (*n* = 7). The relative fluorescence intensity was calculated using 1 − (A × I_b_/I_s_) (I_s_: mean light intensity of selected fluorescence signal area, A: total pixel number of selected fluorescence signal area, and I_b_: mean of background intensity selected area). Plt: platelet, WB: whole blood, ADP: adenosine di-phosphate), NS indicates no significant difference; ** indicates *p* < 0.01, and *** indicates *p* < 0.001.

DiOC6-labeled samples were captured, and their relative fluorescence intensities were compared. Their relative fluorescence intensities were 0.012 ± 0.005, 0.021 ± 0.011, 0.072 ± 0.023, and 0.168 ± 0.075 um. The relative intensity varied with the addition of ADP; in particular, the relative intensity of whole blood with ADP aggregation was much higher than that of platelet-poor blood (Figure 6). This experimental result confirmed the existence of platelet aggregation in whole blood. Increased aggregation was observed with the addition of ADP, which indirectly affected speckle size changes.

## 4. Discussion

The results of our experiment indicate that the speckle size of whole and platelet-poor blood differs using a custom-designed microfluidic channel. Sample aggregation affects speckle size changes based on the speckle contrast and signal intensity obtained after passing through a sample [23,24]. At low concentrations and weak scattering, the speckle size increased. Moreover, at the highest concentration with strong scattering, the speckle size increased [22,33]. Particle size, viscosity, and flow rate are interrelated; as the size and viscosity of particles increase, the flow rate decreases [7]. Figure 4 shows evidence of increases in the speckle size evolution with the variance in particle concentration, exhibiting a tendency similar to that obtained in previous research [34,35].

For platelet-poor blood samples, almost all platelets were removed using the centrifugation method [36]. Using flow cytometry, we confirmed that the platelet number affected the speckle size variation between whole and platelet-poor blood. We showed the difference between the platelet number of whole and platelet-poor blood and the correlation between speckle size and platelet counts (Figure 4). However, platelet-poor blood, which was prepared by removing the buffy coat region and platelets, exists in the buffy coat region, and the effect of these mononuclear cells on the speckle size remains unknown by confirming only the number of platelets.

We also used an ADP agonist to activate platelets to measure the speckle size [37]. The speckle size variation is shown in Figure 4c,d. The addition of an ADP agonist to whole blood resulted in an increased speckle size compared to whole blood without agonists, platelet-poor blood, and platelet-poor blood with ADP. In contrast, platelet-poor blood with ADP had a speckle size similar to that of platelet-poor blood without ADP. The ADP agonist potentially affects the activation of platelets in whole blood to various degrees.

The speckle size was determined by the intensity profile; for weak scattering, the speckle size decreased linearly with scattering [23]. According to previous research, the speckle size is related to the speckle contrast; at high contrast, the speckle size increases [38]. Speckle contrast values range between 0 and 1, where 1 represents no motion and 0 indicates the fastest motion required to blur all speckles. Speckle contrast increases as blood flow speed decreases, and blood flow speed decreases as blood clotting increases [22,39,40,41]. Thus, we demonstrated that the speckle contrast increased as the blood flow speed decreased, as evidenced by the platelet aggregation on the microfluidic aggregates of the microfluidic chips from the DIOC6 staining method in Figure 5 and Figure 6. This result is similar to those reported in [41,42]. However, there may be other variables that must be considered, such as the optical thickness, environmental conditions, and optical setup, in addition to a decrease in the internal speed of the flow chip owing to the aggregation of platelets.

We demonstrated that the determination of the laser speckle size with an optofluidic system can provide a quantitative testing method of platelet function using the platelet number. Our technique is cheaper than previous systems, easy to control, requires a small sample volume (20 µL), and only takes less than 30 s. Our results indicate that speckle size variation based on the platelet number could provide a rapid and simple measurement for screening bleeding disorders in the presurgical or preoperative system. However, while our technique shows promise in detecting variations in speckle size, which correlates with platelet functionality, the real test of its clinical relevance lies in its ability to distinguish these variations accurately in patient samples under real-world conditions. Future work should focus on refining the technique to enhance its diagnostic precision and reliability in clinical settings, potentially including a broader range of platelet concentrations and conducting prospective clinical trials to validate its effectiveness.

## 5. Conclusions

We demonstrated the speckle size variation between whole and platelet-poor blood using a simple dynamic microfluidic system that provides an assay for platelet function test devices. The speckle size of each sample obtained through the microchannel was used as the standard for platelet function. The speckle size showed a strong dependence on the whole and platelet-poor blood conditions. Our findings indicate that the proposed microfluidic system provides faster and simpler processes. Therefore, the device can be used as a diagnostic tool for individual blood samples.

## Data Availability

The data that support the findings of this study are available from the corresponding author upon reasonable request.
